# Scoping review about pathogenesis, risk factors, and treatment of venous and arterial thrombosis in coronavirus infection

**DOI:** 10.3389/fcvm.2025.1688115

**Published:** 2025-12-12

**Authors:** Diana Malaeb, Sara Mansour, Nada Dia, Nada M. Kassem, Chadia Haddad, Mariam Dabbous, Ola Ismail, Farah Adel, May Gamal, Jisha Myalil Lucca, Sami El Khatib, Pascale Salameh, Souheil Hallit, Hassan Hosseini

**Affiliations:** 1 College of Pharmacy, Pharmacy Practice, Gulf Medical University, Ajman, United Arab Emirates; 2 School of Pharmacy, Lebanese International University, Beqaa Governorate, Lebanon; 3 INSPECT-LB (Institut National de Santé Publique, d'Épidémiologie Clinique et de Toxicologie-Liban), Beirut, Lebanon; 4 Faculty of Public Health, Lebanese University, Fanar, Lebanon; 5 Research Department, Psychiatric Hospital of the Cross, Jal Eddib, Lebanon; 6 Inserm U1094, IRD UMR270, EpiMaCT Epidemiology of Chronic Diseases in Tropical Zone, University of Limoges, Limoges, France; 7 Department of Biological Sciences, School of Arts and Sciences, Lebanese International University, Bekaa, Lebanon; 8 Center for Applied Mathematics and Bioinformatics (CAMB), Gulf University for Science and Technology, Hawally, Kuwait; 9 Faculty of Pharmacy, Lebanese University, Hadath, Lebanon; 10 Department of Primary Care and Population Health, University of Nicosia Medical School, Nicosia, Cyprus; 11 School of Medicine, Lebanese American University, Byblos, Lebanon; 12 School of Medicine and Medical Sciences, Holy Spirit University of Kaslik, Jounieh, Lebanon; 13 Applied Science Research Center, Applied Science Private University, Amman, Jordan; 14 UPEC-University Paris-Est, Créteil, France; 15 RAMSAY SANTÉ, HPPE, Champigny-sur-Marne, France

**Keywords:** COVID-19, thrombosis, venous thromboembolism, arterial thrombosis, myocardial infarction, stroke, deep vein thrombosis, pulmonary embolism

## Abstract

**Introduction:**

Coronavirus disease 2019 (COVID-19), caused by SARS-CoV-2, is now understood as a systemic illness marked by a distinctive coagulopathy that extends beyond its primary respiratory manifestations. Direct viral injury to the endothelium and an exaggerated inflammatory “cytokine storm” and complement activation disrupt normal hemostasis and create a prothrombotic environment. This scoping review aims to synthesize and compare the mechanisms, risk factors, and antithrombotic strategies associated with venous and arterial thrombosis in COVID-19.

**Methods:**

A scoping review of English-language studies indexed in PubMed/Medline, OVID, and Wiley Library was conducted from January 2020 to June 2024. Search terms related to COVID-19, thrombotic complications, pathophysiological mechanisms, and antithrombotic therapies were included. Clinical trials, cohort and retrospective observational studies, systematic reviews, meta-analyses, and case reports are included. Two reviewers independently screened titles, abstracts, and full texts for relevance and extracted data to map current evidence on venous and arterial thrombosis in COVID-19.

**Results:**

COVID-19-related coagulation problems can cause both venous and arterial thrombosis. Venous thromboembolism, which includes deep vein thrombosis and pulmonary embolism, occurs in about 4% to 15% of hospitalized patients and can increase to 30% in those in intensive care, even with standard prevention. Elevated D-dimer levels are strongly associated with a higher risk of clot formation. Arterial clots, like strokes or heart damage, are less common but generally more serious, caused by platelet activation, inflammation, and small vessel blockage rather than just slow blood flow in veins. Evidence indicates that low-molecular-weight heparin is the preferred anticoagulant because it reduces both inflammation and clotting. Therapeutic doses may be especially beneficial for high-risk patients, and continuing clot prevention after hospital discharge helps lower the risk of late clots without significantly increasing bleeding risk.

**Conclusion:**

Recognition of COVID-19–associated coagulopathy underscores the necessity of early risk stratification and individualized anticoagulation to mitigate thrombotic events and improve outcomes. Extended post-discharge prophylaxis appears promising in reducing late thrombotic complications. Future research should aim to refine optimal anticoagulant regimens and determine ideal prophylaxis duration for COVID-19–related thrombosis to reduce morbidity and mortality rates.

## 1 Introduction

Coronavirus disease 2019 (COVID-19), caused by the severe acute respiratory syndrome coronavirus 2 (SARS-CoV-2), has been linked to severe inflammatory responses and a hypercoagulable state, leading to multiple thrombotic complications. COVID-19 has resulted in a global pandemic, with more than 778 million confirmed cases and over 7.1 million deaths worldwide to date (1). COVID-19 is diagnosed based on a positive COVID-19 polymerase chain reaction or antigen test in various clinical settings. Severe COVID-19 infection is identified as oxygen saturation <94% on room air, a respiratory rate >30 breaths per minute, or lung infiltrates >50% (2, 3). The virus's primary target is the respiratory tract, producing a range of clinical manifestations from mild upper respiratory symptoms to severe pneumonia accompanied by life-threatening respiratory distress syndrome (ARDS). However, COVID-19 is now recognized as a multisystem disease capable of affecting the cardiovascular, renal, neurological, and hematological systems, causing diverse and sometimes life-threatening complications (4–6). In particular, COVID-19 is also associated with cardiac issues, including right ventricular dysfunction and pulmonary hypertension, which may worsen the severity of pulmonary embolism and negatively impact cardiovascular outcomes (7). Over time, SARS-CoV-2 has evolved into multiple variants, including Alpha, Beta, Gamma, Delta, and Omicron, with newer Omicron sublineages such as XBB and EG.5 showing increased transmissibility and immune escape potential, which may also influence disease severity and thrombotic risk (8).

Both venous thromboembolism (VTE), including deep vein thrombosis and pulmonary embolism, and arterial thrombosis, such as myocardial infarction, ischemic stroke, and limb ischemia, are recognized as significant complications of COVID-19, resulting from a combination of hyperinflammation, endothelial injury, and coagulation activation. SARS-CoV-2 binds to ACE2 receptors on endothelial cells, causing dysfunction and endotheliitis that promote platelet adhesion and coagulation, cytokine storms, neutrophil extracellular traps (NETs), and complement activation (9–11). Interestingly, similar inflammatory and endothelial pathways are observed in viral infections such as influenza, cytomegalovirus, and HIV, which can also trigger coagulation activation and thrombotic complications. However, the higher intensity and persistence of endothelial dysfunction and immunothrombosis seen in COVID-19 may explain its greater thrombotic burden than other viruses (12, 13).

These thrombotic events significantly contribute to multi-organ failure and increased mortality among hospitalized COVID-19 patients, underscoring the crucial role of coagulopathy in disease severity and outcomes (14). The incidence of thromboembolic events in COVID-19 patients varies widely among studies, ranging from 3.9% to 14.7% in hospitalized populations (3, 10, 15, 16). The actual burden of venous thromboembolism (VTE) may be underestimated, as pulmonary embolism (PE) can cause sudden respiratory decompensation, which is often misattributed to severe COVID-19 infections. Such variability can arise from differences in VTE prophylaxis protocols, screening strategies, patient selection, and reporting bias, leading to efforts like multiple meta-analyses aimed to better estimate its prevalence (17). A meta-analysis involving 20 studies and 1,988 hospitalized COVID-19 patients reported an average VTE rate of 31.3%, with similar rates observed in intensive care unit (ICU) patients (32.7%) (18).

## 2 Objectives

Thrombosis is a common complication of COVID-19, but most research focuses only on either arterial or venous events, leaving a gap in understanding their differences and similarities. Comparing these types of thrombosis can help clarify their distinct pathophysiological mechanisms, risk factors, and outcomes while identifying shared pathways like endothelial injury, inflammation, and hypercoagulability. This scoping review mainly aims to map and synthesize existing evidence on the pathogenesis, risk factors, and management of arterial and venous thrombosis in patients with COVID-19. The secondary objectives are to explore and describe differences and overlaps in their underlying mechanisms and clinical manifestations, identify key knowledge gaps to guide future research, and enhance clinical understanding of COVID-19-related thrombotic complications.

## 3 Materials and methods

### 3.1 Study framework

This study was conducted as a scoping review following the Preferred Reporting Items for Systematic Reviews and Meta-Analyses extension for Scoping Reviews (PRISMA-ScR) guidelines (19).

### 3.2 Search strategy

A literature search was performed using PubMed/MEDLINE, Embase, Wiley Online Library, and Ovid from January 2020 to September 2024. A well-structured search strategy combined relevant keywords and medical subject headings (MeSH terms). The main search terms included COVID-19 related words such as “COVID-19,” “coronavirus disease,” and “SARS-CoV-2,” which were paired with additional terms. For thrombosis complications, terms like “acute respiratory distress syndrome,” “pulmonary embolism,” “coagulation,” “venous thromboembolism,” “arterial thrombosis,” “stroke,” and “myocardial infarction” were added. For the pathophysiology, terms were limited to “pathophysiology of COVID-19,” “cytokine storm,” “endothelial dysfunction,” “angiotensin-converting enzyme 2 (ACE2),” and “immunothrombosis.” Concerning treatment, terms such as “antithrombotic therapy,” “anticoagulants”, “heparin”, “low-molecular-weight heparin”, and “antiplatelet therapy” were used.

### 3.3 Eligibility criteria

The search was restricted to articles published in English, including case reports, clinical trials, systematic reviews, meta-analyses, cohort studies, journal articles, retrospective observational studies, epidemiological statistics, and randomized controlled trials. Studies focusing solely on non-COVID-19 thrombosis, animal models, or without relevant clinical data were excluded.

### 3.4 Study selection

Initial screening was performed based on titles and abstracts to determine relevance to the review's focus. Articles that met the initial inclusion criteria were selected for full-text review. A two-step review was conducted with an initial assessment of titles and abstracts to filter out irrelevant studies, followed by a detailed evaluation of the full-text articles to confirm their eligibility.

### 3.5 Data extraction and charting

The included studies systematically extracted relevant data, including study design, participant demographics, sample size, type of thrombotic event (arterial or venous), underlying pathophysiological mechanisms, identified risk factors, treatment regimens, and clinical outcomes such as complications or mortality. Later, the extracted data were synthesized to provide a comprehensive overview of the current evidence on thrombosis in COVID-19, highlighting differences and similarities between arterial and venous events regarding pathogenesis, risk factors, and management strategies.

## 4 Results

### 4.1 Study selection

**Figure 1:**
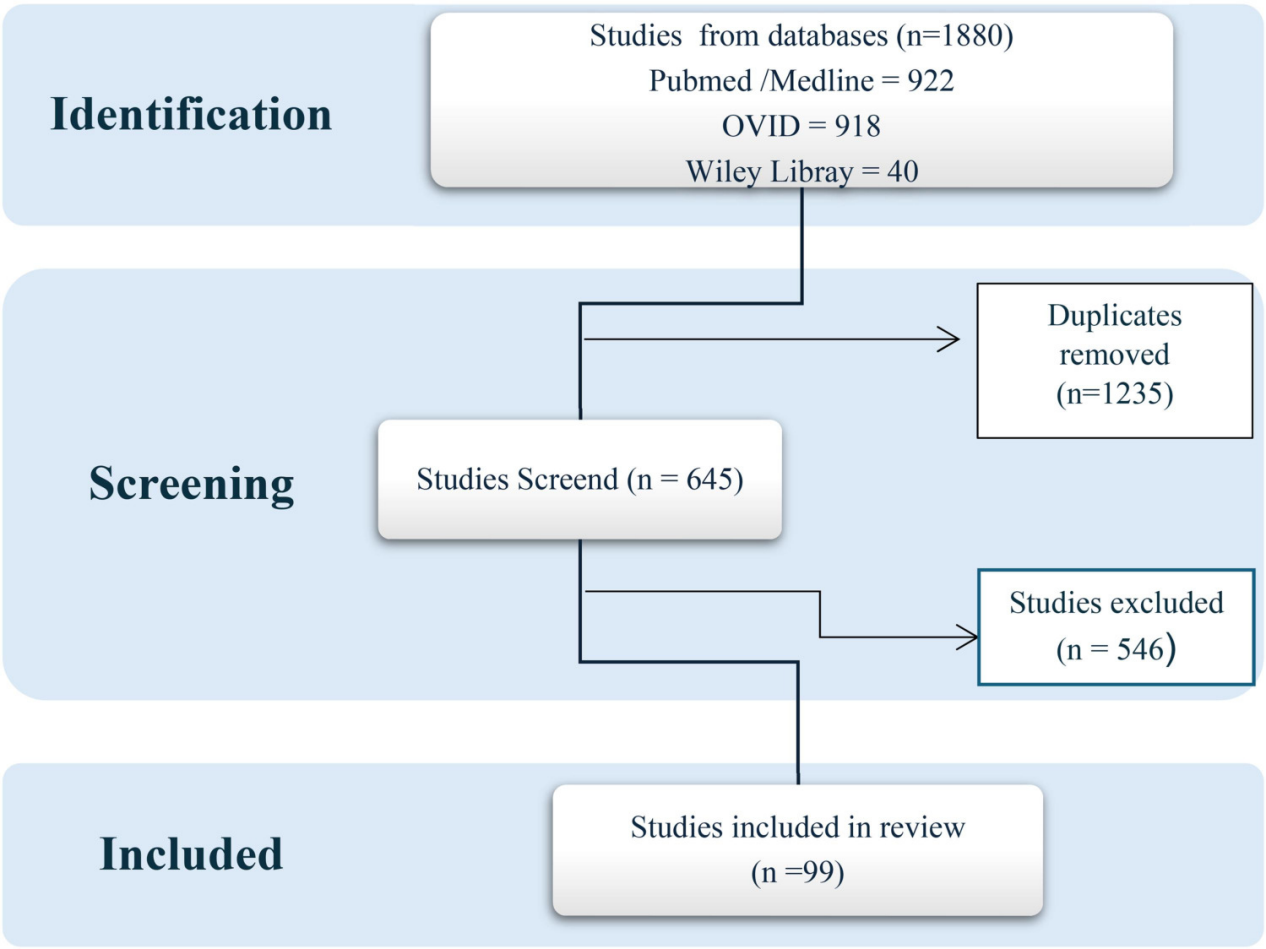
Study identification PRISMA flow diagram.

The initial search across three databases retrieved 1,880 records (PubMed: 922; Ovid: 918; Wiley Library: 40). After removing 1,235 duplicates, 645 unique articles remained for title and abstract screening. Following this process, 120 full-text articles were assessed for eligibility. 99 studies met the inclusion criteria and were included in the final scoping review. Figure 1 (PRISMA flow diagram) outlines the study identification, screening, eligibility assessment, and inclusion process.

### 4.2 Study characteristics

This scoping review included 88 studies, published from 2020 to 2024, reflecting the rapid development of knowledge about thrombosis in COVID-19. Most of the included studies were observational, including retrospective and prospective cohort studies and case–control analyses. Additionally, several systematic and narrative reviews examined various aspects of venous and arterial thrombotic complications related to COVID-19 infection.

Research originated from Asia, particularly China, with substantial contributions from Europe(Italy, the United Kingdom, and Spain), and North America, mainly the United States. These regions were among the most severely impacted during the early stages of the pandemic. The sample sizes varied widely, from small studies with fewer than 20 patients experiencing severe COVID-19-related thrombosis to large multicenter investigations involving thousands of hospitalized patients. While most studies focused on hospitalized adults with moderate to severe illness, some also examined outpatients and post-discharge groups, offering insights into longer-term thrombotic risks.

Throughout the literature, researchers examined a wide range of outcomes. These included the incidence and factors associated with venous thromboembolism (VTE), deep vein thrombosis (DVT), and pulmonary embolism (PE); arterial events such as myocardial infarction, ischemic stroke, and peripheral arterial thrombosis; biochemical markers of coagulopathy (notably D-dimer, fibrinogen, and platelet count); and antithrombotic management strategies, including heparin dosing, direct oral anticoagulant use, and prophylaxis duration. A concise summary of each included study, highlighting publication details, design, and key findings, is provided in Supplementary Table S1.

### 4.3 Clinical signs and symptoms of COVID-19

#### 4.3.1 Incubation period and progression

Typically, the incubation period of COVID-19 ranges from 4 to 5 days, with the likelihood of symptom development even after 14 days of exposure. A change in duration is expected based on the infecting variant and patient factors (20). During this period, also known as the “presymptomatic” period, infected individuals can still spread the virus. This means they might infect others before they even start developing clinical symptoms (21).

#### 4.3.2 Stages of COVID-19

According to the Matricardi model, COVID-19 can be categorized into four distinct stages, each characterized by unique clinical features and severity (15). The early infection stage (Stage 1) typically presents mild upper respiratory symptoms such as sore throat, cough, fever, myalgia, malaise, and occasional gastrointestinal involvement. As the disease advances into the pulmonary phase (Stage 2), viral spread to the lower respiratory tract results in more pronounced respiratory manifestations, often leading to dyspnea and bilateral pneumonia visible on imaging. In the hyperinflammatory phase (Stage 3), excessive immune activation and cytokine release trigger severe complications such as acute respiratory distress syndrome (ARDS), cardiac and renal injury, sepsis, and secondary infections may develop, requiring intensive care and close monitoring. The final stage (stage 4) determines the clinical outcome, recovery or death, often influenced by thromboembolic events, disseminated intravascular coagulation (DIC), and multiorgan failure. Mortality is strongly associated with advanced age, comorbidities, respiratory failure, high D-dimer levels, lymphopenia, and severe systemic inflammation (10, 22–25).

### 4.4 Pathogenesis of thrombosis in COVID-19

#### 4.4.1 General pathway

**Figure 2:**
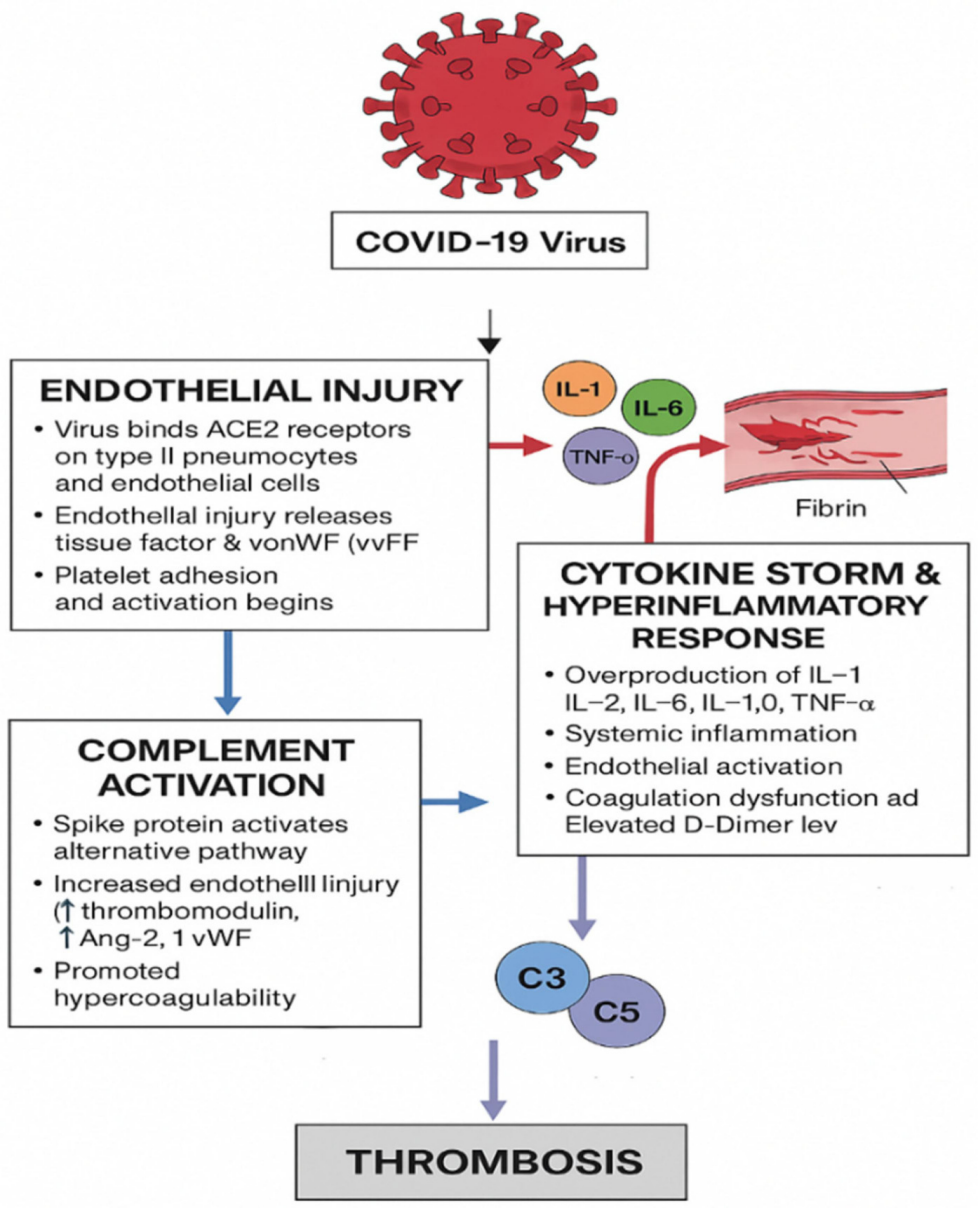
Proposed mechanisms of COVID-19–associated thrombosis involving endothelial injury, inflammation, and complement activation. The authors created this figure to summarize findings from included studies.

The cascade of events in coagulopathy in COVID-19 is interconnected by viral infection, endothelial injury, and systemic inflammation, which leads to a hypercoagulable state and increased risk of thromboembolic complications (26). One leading hypothesis underlying the mechanism of thrombosis is that when the body attacks SARS-CoV-2, it overproduces signaling molecules (a “cytokine storm”), which damages the inner lining of blood vessels. That damage releases tissue factors, making the blood more prone to clotting and raising the chance of developing venous blood clots (27). The proposed mechanisms of COVID-19–associated thrombosis are illustrated in Figure 2.

##### 4.4.1.1 Direct viral attack on endothelial cells

In the early stage of infection, COVID-19 invades host cells via its spike (S) protein and directly attacks endothelial cells, leading to cellular injury that activates both the primary and secondary pathways of hemostasis (28). The SARS-CoV-2 interacts with the type II pneumocytes through several membrane proteins, including the membrane-bound angiotensin-converting enzyme 2. The viral spike protein binds to angiotensin-converting enzyme 2 (ACE2) receptors expressed on type II pneumocytes and endothelial cells (29). This endothelial injury promotes the release of pro-coagulant mediators, including tissue factor and von Willebrand factor (vWF), which enhance platelet adhesion and activation, initiating thrombus formation.

##### 4.4.1.2 Cytokine storm and hyper-inflammatory response

Following viral entry, an exaggerated immune response develops, characterized by the release of pro-inflammatory cytokines, including interleukin-1 (IL-1), interleukin-6 (IL-6), interleukin-2 (IL-2), interleukin-10 (IL-10), and tumor necrosis factor-alpha (TNF-α) (30). This excessive cytokine production, often termed a “cytokine storm,” induces systemic inflammation, endothelial activation, and increased vascular permeability. Elevated D-dimer levels and widespread fibrin deposition indicate coagulation dysfunction and the development of a hypercoagulable state (31, 32).

##### 4.4.1.3 Complement system activation

The SARS-CoV-2 spike protein also triggers the activation of the alternative complement pathway, further amplifying vascular injury and inflammation. Activation of complement components C3 and C5 generates C3a and C5a, potent inflammatory mediators that promote endothelial damage, leukocyte recruitment, and cytokine release. C5b contributes to the formation of the membrane attack complex (MAC), directly injuring endothelial cells (33). Elevated levels of complement-related markers such as von Willebrand factor, thrombomodulin, and angiopoietin-2 are frequently observed in patients with severe COVID-19, underscoring the link between complement activation, endothelial dysfunction, and thrombotic complications (34, 35).

#### 4.4.2 Venous thrombosis pathway

Venous thrombosis in COVID-19 primarily results from the classical Virchow's triad, which includes endothelial injury, venous stasis, and hypercoagulability. Infected patients often experience prolonged immobilization, hypoxia, and systemic inflammation, all of which promote venous stasis and raise thrombotic risk. The direct viral invasion of endothelial cells via ACE2 receptors causes vascular injury and upregulation of tissue factor, encouraging platelet adhesion and activation.

Additionally, hypoxia triggers the production of hypoxia-inducible factor-1α (HIF-1α) and plasminogen activator inhibitor-1 (PAI-1), suppressing fibrinolysis and promoting clot persistence. The cytokine storm seen in severe COVID-19—involving elevated levels of interleukins (IL-6, IL-1β) and tumor necrosis factor-α (TNF-α)—increases thrombin generation and fibrin deposition. Moreover, complement activation and neutrophil extracellular traps (NETs) further boost coagulation signaling.

These interconnected mechanisms ultimately lead to fibrin-rich thrombi forming within the deep venous system and pulmonary vasculature, explaining the high occurrence of deep vein thrombosis (DVT) and pulmonary embolism (PE) (2, 9, 12, 29).

#### 4.4.3 Arterial thrombosis pathway

Arterial thrombosis in COVID-19 mainly arises from endothelial dysfunction, platelet hyperreactivity, and inflammation-induced coagulation activation. This virus's direct invasion of endothelial cells causes widespread endothelialitis, which disrupts vascular integrity and leads to increased permeability, decreased nitric oxide production, and the loss of natural anticoagulant properties. The subsequent downregulation of ACE2 results in excess angiotensin II, promoting vasoconstriction, oxidative stress, and further endothelial damage (12).

The ensuing cytokine storm, mainly driven by IL-6, IL-8, and TNF-α, increases the expression of intercellular adhesion molecule-1 (ICAM-1) and vascular cell adhesion molecule-1 (VCAM-1), promoting leukocyte adhesion and local vascular inflammation. Activated platelets release platelet factor 4 (PF4), thromboxane A_2_, and adenosine diphosphate (ADP), potent mediators that enhance platelet aggregation and thrombin production (5, 31).

**Figure 3:**
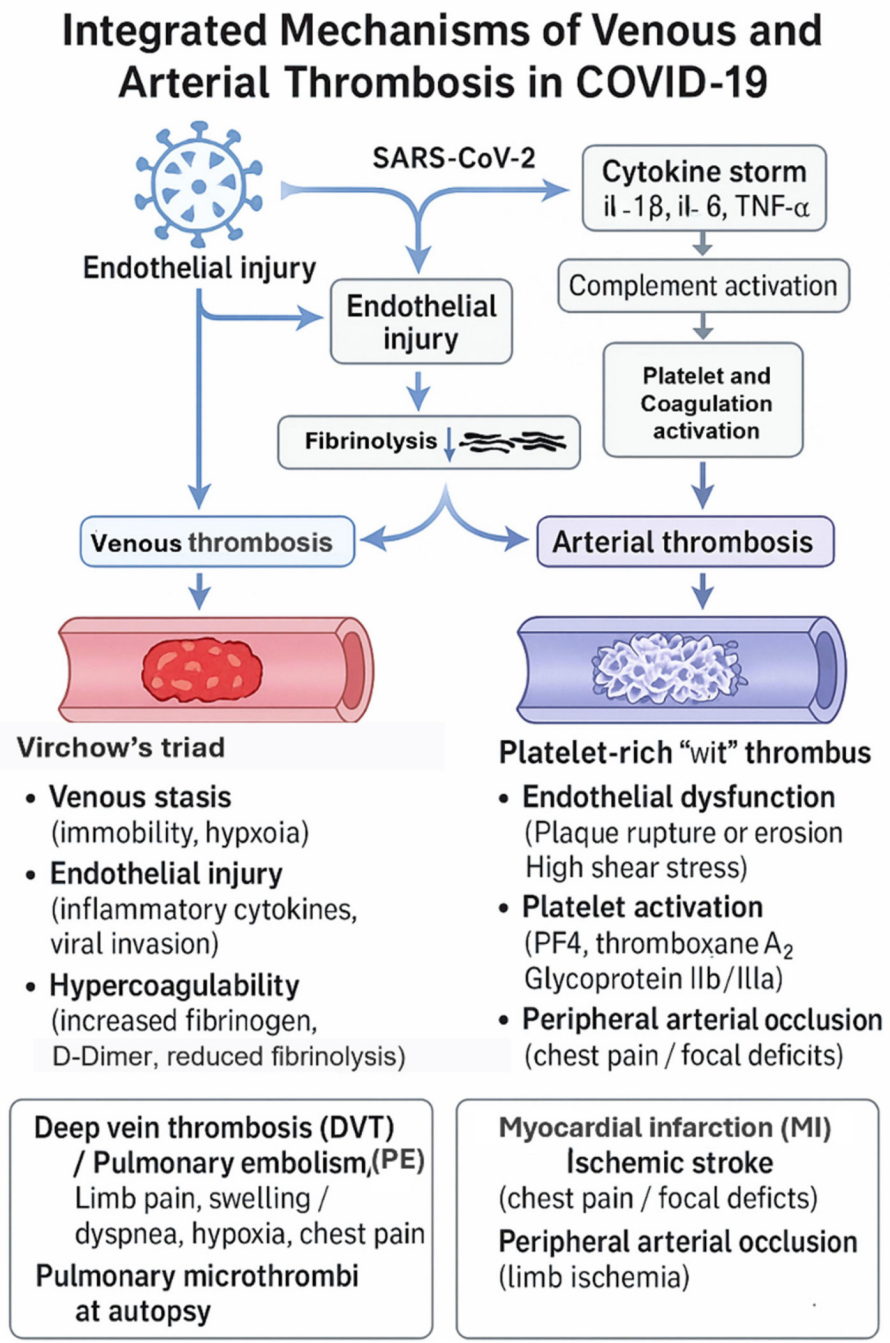
Differentiated mechanisms of fibrin-rich (venous) and platelet-rich (arterial) thrombus formation in COVID-19. The authors created this conceptual figure to summarize findings from included studies.

These synergistic mechanisms form platelet- and fibrin-rich microthrombi within arterial vessels, especially in the coronary, cerebral, and renal arteries. Clinically, this accounts for the higher rate of myocardial infarction, ischemic stroke, and other arterial thrombotic complications seen in patients with severe COVID-19 infection (2, 5). Venous and arterial thrombotic mechanisms in COVID-19 are summarized in Figure 3.

### 4.5 Risk factors

Critically ill COVID-19 patients are at risk of thromboembolic events due to several factors, such as the exaggerated inflammatory response, prolonged hypoxia, immobilization, and the development of disseminated intravascular coagulation (DIC). These conditions together contribute to an increased risk of both venous and arterial thromboembolism (36). Recently, studies have identified a concerning association between the COVID-19 vaccine and the onset of Vaccine-Induced Immune Thrombotic Thrombocytopenia (VITT). VITT is a rare but severe condition characterized by the formation of antibodies against platelet factor 4 (PF4) that is bound to platelets. These antibodies trigger excessive platelet activation, which, in turn, stimulates the coagulation cascade. This hyperactivation of the coagulation system leads to clinically significant thromboembolic complications, manifesting in both venous and arterial systems (37).

### 4.6 Thrombotic manifestations and prognosis

Patients with coronavirus infections often have macrovascular or microvascular thrombosis associated with poor prognosis (38). Respiratory viruses can influence all coagulation cascade components, including primary hemostasis, fibrinolysis, and coagulation, which are linked to increased incidence of DVT and PE (39). The true prevalence of thrombosis in people infected with COVID-19 is unclear. The level of the illness and the type of ward hospital admission impact the level of anticoagulation needed and will affect future thrombosis. Unlike most critically ill patients, venous thromboembolism occurs in COVID-19 patients despite thromboprophylaxis (38, 40). A cross-sectional study conducted in Pakistan on confirmed COVID-19 cases revealed that patients who develop thrombosis have higher mortality, especially among males and those aged beyond 56 years. The study also identified pulmonary embolism with COVID-19 infection as a poor prognostic indicator (41). Thus, in severe COVID-19 infections, systemic inflammation and coagulopathy occur with four main thrombotic disorders: myocardial injury, pulmonary embolism, deep vein thrombosis, and stroke**.**

#### 4.6.1 Arterial thrombotic manifestations

##### 4.6.1.1 Myocardial injury (MI)

According to the patient's clinical characteristics, myocardial involvement can range from viral myocarditis to atherothrombotic myocardial infarction (42, 43). Regrettably, during the pandemic, patients with acute coronary syndrome (ACS) experienced delayed or avoided presentation to acute care settings; these delays were driven mainly by increased concern of being infected by SARS-CoV-2 in the hospital and efforts to follow physical distancing guidelines. In patients with severe COVID-19, rapid clinical decline often prevents completion of standard diagnostic tests, making risk assessment and treatment decisions even more difficult (44).

Approximately 25% of patients hospitalized with COVID-19—including those without prior cardiovascular disease—experience myocardial injury, with incidence rising to 55 percent among individuals with pre-existing cardiovascular conditions. The viral infection in the heart could affect the myocardial and vascular endothelial cells, resulting in myocarditis (44, 45).

Also, the virus could trigger inflammation of blood vessel walls, leading to myocardial vasculitis (46). In addition, a microvascular injury may result from thrombosis in the myocardial microcirculation with normal epicardial coronary vessels (44, 47–50).COVID-19 patients who develop a hyperinflammatory state may advance to stress cardiomyopathy and multiple organ failure due to the intense release of potent inflammatory cytokines and microvascular damage (46, 48–50). Physical stress can cause stress-induced cardiomyopathy, sometimes referred to as Takotsubo cardiomyopathy (TCM), which has a striking clinical appearance that typically resembles an abrupt myocardial infarction. A greater risk of acute coronary events has been linked to pneumonia hospitalization (50). While it is likely that the COVID-19 pandemic increases the number of atherothrombotic cases, there is no evidence of a global increase in myocardial infarction cases. Furthermore, as pneumonia due to COVID-19 is associated with hypoxemia, sepsis, septic shock, and coagulopathy, it can impair coronary perfusion in severe cases (50). Cytokine storm-induced sympathetic activation and biological stress may trigger coronary vasospasm. All these factors will disrupt the oxygen supply/demand balance, resulting in myocardial damage with non-obstructive pulmonary disease. Direct viral invasion is not the only mechanism by which SARS-CoV-2 can damage the heart. A particularly interesting interaction has been described between SARS-CoV-2 and the renin angiotensin system (RAS), which increases the activation of Angiotensin II Receptor Type I(AT1), triggering vasoconstriction with significant effects on endothelium, inflammation, and coagulation, raising vascular permeability and precipitating organ damage. It is reported that COVID-19 patients have initially elevated Ang II levels at cardiac pericytes, endothelium of the arteries and veins in the circulatory system (44).

In COVID-19–associated thrombosis, myocardial injury can result not only from viral myocarditis or systemic inflammation but also as a consequence of acute right heart strain secondary to massive pulmonary embolism (PE). The abrupt increase in pulmonary vascular resistance caused by large thromboemboli leads to elevated right ventricular (RV) afterload, dilation, and dysfunction, which may progress to right heart failure and cardiogenic shock (7, 51).

Clinically, these cardiac complications present significant challenges, as diagnostic testing is often delayed or incomplete due to rapid deterioration or infection control constraints. Although COVID-19 has not caused a global increase in myocardial infarction incidence, affected patients, particularly the elderly and those with cardiovascular comorbidities, exhibit a higher risk of adverse outcomes, prolonged hospitalization, and mortality (52–55).

#### 4.6.2 Venous thrombosis manifestations

##### 4.6.2.1 Pulmonary embolism (PE)

Despite thromboprophylaxis, pulmonary embolism was reported in critically ill COVID-19 patients. Pulmonary embolism causes impaired gas exchange due to pulmonary vascular obstruction, leading to a mismatch in the ventilation/perfusion ratio because alveolar ventilation remains the same. In contrast, pulmonary capillary blood flow effectively decreases, causing dead space ventilation and hypoxemia (15).

Liao et al. reviewed 19 studies, and the pooled incidence of pulmonary embolism in patients with COVID-19 was 15.3% (56). Roncon et al. identified 23 studies and found that the pooled in-hospital PE rate was 23.4% in COVID-19 patients in the ICU and 14.7% in general wards (57).

A study evaluating 413 patients with COVID-19 stated that males, a history of smoking, increased levels of D-dimer, elevated lactate dehydrogenase (LDH), and low ferritin are risk factors for PE in COVID-19. In addition, D-dimer levels above 1600 ng/mL can help predict PE with a sensitivity of 100% and a specificity of 62% (58). Two more small studies showed that the incidence rate of VTE in the first 30–42 days after hospitalization due to COVID is 0.6%–0.48% (59, 60).

The clinical status of the patients has been strongly correlated with PE incidence, with patients in intensive care presenting a higher risk than non-hospitalized patients. Furthermore, anti-inflammatory and prophylactic antithrombotic therapy may affect the incidence (29). A study assessed the incidence and severity of pulmonary embolism in COVID-19 infections in different variants, alpha, omicron, and delta. The findings regarding the association between vaccine administration and the development of thrombosis are controversial. In one study, 10% of vaccinated patients developed PE instead of 7% of unvaccinated patients. In another study enrolling 884,828 vaccinated patients against BNT162b2 mRNA COVID-19, the risk of pulmonary embolism was lower with the vaccine than with the control group (61). However, vaccines have proven highly effective in preventing infection, halting disease progression, decreasing hospitalization, and minimizing death. Other relevant studies show that the risk of PE after COVID-19 infection is still high up to 110 days post-infection (3, 15, 62). Alterations in the coagulation cascade mainly cause thrombotic events not associated with COVID-19 because of coagulation deficiency or specific risk factors. In thrombotic events related to COVID, there are no alterations in coagulation factors or the presence of thrombophilia in PE (63). The hypercoagulable state of COVID-19 is mainly due to a unique derangement in the hemostatic pathways. This viral infection's constant stimulation of endothelial cells, platelets, and other inflammatory cells promotes the upregulation of procoagulant factors. It destroys the vascular endothelium's protective function, causing abnormal coagulation (64).

##### 4.6.2.2 Deep vein thrombosis (DVT)

Excessive inflammation, platelet activation, endothelial dysfunction, and stasis, the COVID-19 virus can predispose patients to thrombotic disease in both the venous and arterial circulation (40). A high rate of asymptomatic DVT (14.7%) was found among COVID-19 patients with pneumonia admitted to non-intensive care units (65). A prevalence of 46.1% of DVT was found in a study done among COVID-19 patients (66). A retrospective cohort study on COVID-19 patients showed increased incidence of DVT aggravation in the cases compared to the control group, 44.62% vs. 17.86% respectively (54). There are differences in the locations of DVT in patients. According to several studies, the most common is the unilateral or bilateral leg DVT (67). Additionally, there is a strong correlation between the length of hospital stay resulting from COVID-19 and the risk of DVT. On days 7, 14, and 21, for instance, the prevalence of DVT was 16%, 33%, and 42%, respectively. This is most probable because patients in the hospital for an extended period have several COVID-19 problems and severity (15). In hospitalized COVID-19 patients, studies have revealed that DVT is correlated with adverse outcomes such as more ICU admissions, fewer hospital discharges, more deaths, and lower actuarial survival rates (66).

### 4.7 Treatment and management of thrombosis in COVID-19

#### 4.7.1 General therapeutic strategies

The management of thrombosis in patients with COVID-19 is multifaceted, aiming to address the overlapping processes of viral injury, inflammation, endothelial dysfunction, and hypercoagulability underlying arterial and venous events. Patients with pre-existing chronic diseases should continue their baseline therapies unless contraindicated or where significant drug interactions are expected. In all cases, treatment decisions must balance the benefits of thrombosis prevention against bleeding risk and the potential for drug–drug interactions (68, 69).

Hospitalized COVID-19 patients are at particularly high risk for venous thromboembolism (VTE), including deep vein thrombosis and pulmonary embolism, due to immobility, systemic19 inflammation, and endothelial injury. For this reason, routine thromboprophylaxis using low-molecular-weight or unfractionated heparin is strongly recommended for most inpatients, alongside mechanical measures when appropriate (70). Therapeutic-dose anticoagulation may be justified in individuals with markedly elevated D-dimer levels or evidence of ongoing coagulopathy. Findings from the HEP-COVID trial demonstrated that therapeutic-dose heparin significantly reduced the combined outcome of venous and arterial thromboembolic events and death compared with prophylactic dosing. However, overall mortality remained similar by day 32 (71, 72).

Meanwhile, arterial thrombosis, presenting as myocardial infarction, ischemic stroke, or peripheral arterial occlusion, necessitates a comprehensive approach that includes both anticoagulant and antiplatelet therapies alongside targeted cardiovascular care. The standard treatment of acute coronary syndromes adheres to established cardiology guidelines, with primary percutaneous coronary intervention (PCI) being the preferred method for ST-elevation myocardial infarction when prompt access is available. If immediate PCI is unavailable, fibrinolytic therapy may be an option. Patients experiencing ischemic stroke due to large-vessel occlusion might benefit from thrombolysis with tissue plasminogen activator (tPA) or mechanical thrombectomy, as long as no contraindications are present (72, 73).

Anti-inflammatory and antiviral treatments can also be supported by reducing endothelial inflammation and cytokine-driven coagulation activation. Nirmatrelvir and Remdesivir have been shown to limit viral replication and may indirectly decrease thrombotic complications (74, 75). The RECOVERY trial demonstrated the survival benefit of dexamethasone (6 mg daily for 10 days) in patients needing oxygen therapy, emphasizing the role of glucocorticoids in reducing cytokine storms that promote thrombosis (76). Adding Tocilizumab or Baricitinib in severe cases may further decrease inflammatory injury and vascular complications (74, 76).

Beyond the acute phase, extended thromboprophylaxis remains an important consideration. A meta-analysis of over 10,000 post-discharge patients found that continuing anticoagulation therapy reduced thrombotic events and all-cause mortality without significantly increasing bleeding risk (77). Such findings support a personalized approach to prophylaxis duration based on D-dimer levels, comorbidities, and mobility status.

Overall, current evidence emphasizes that both arterial and venous thrombosis in COVID-19 share common inflammatory and endothelial pathways but differ in clinical presentation and management intensity. Optimal outcomes depend on timely diagnosis, individualized anticoagulant dosing, integrating antiviral and anti-inflammatory strategies, maintaining adequate oxygenation and hemodynamic stability, and strict glycemic control may limit vascular injury and restore hemostatic balance. Early initiation of rehabilitation and mobilization is strongly encouraged to enhance functional recovery and reduce post-COVID-19 thrombosis disability. Table 1 summarizes the most commonly used medications for the treatment of COVID-19 and associated thrombosis.

**Table 1:** Commonly used medications for treating COVID-19 and associated thrombosis.

Drug	Mechanism	Dosing	Contraindications	Notes
Remdesivir	Antiviral, SARS-CoV-2 RNA-dependent RNA polymerase inhibitor.	200 mg IV infused over 0.5–2 h on day 1, followed by 100 mg IV daily for a maximum duration of 10 days.	Age <12 years? Weight <40 kg, eGFR <30 mL/min.	Laboratory tests needed before initiation are: eGFR, hepatic laboratory testing, and prothrombin time.
Baricitinib	Inhibits the activity of JAK proteins and modulates the signaling pathway of various interleukins, interferons, and growth factors.	BAR dose depends on eGFR; therapy duration is up to 14 days or until hospital discharge, whichever comes first.	eGFR <15 mL/min/1.73 m^2^: Not recommended.	Lipid parameters should be monitored.
Dexamethasone	A glucocorticoid with minimal mineralocorticoid activity.	6 mg PO/IV daily for 10 days or on discharge, whichever comes first.	Age <18 years, systemic fungal infection, allergy, cerebral malaria.	Its use is off-label. The second options are: methylprednisolone, hydrocortisone, and prednisone.
Tocilizumab	Monoclonal antibody, anti-interleukin-6 receptor.	8 mg/kg actual body Weight with a maximum of 800 mg.	Allergy.	Its use is still under investigation. There is a black box warning on severe infections. Regular neutrophils, platelets, lipids, and liver function monitoring are needed.
Heparin (UFH)	Anticoagulant, dose-dependent inhibitor of factors Xa, IX, X, and XII, thrombin formation (factors XI, VIII, V, and fibrinogen).	Dependent on the indication.	History of pentosan polysulfate-induced thrombocytopenia, uncontrolled active bleeding except for DIC, and allergy.	Discontinue at a platelet level of <100,000. Monitor aPTT.
Low Molecular Weight Heparin (LMWH)	Anticoagulant; potentiates antithrombin III inhibition of factor Xa and thrombin.	Prophylactic dose: e.g., enoxaparin 40 mg SC daily; therapeutic dose: 1 mg/kg SC every 12 h. Adjust based on renal function.	Active bleeding, history of heparin-induced thrombocytopenia, severe renal impairment (CrCl <30 mL/min).	Preferred anticoagulant for thromboprophylaxis in hospitalized COVID-19 patients. Monitor anti-Xa levels if renal dysfunction or obesity.
Direct Oral Anticoagulants (DOACs) (e.g., Apixaban, Rivaroxaban, Dabigatran, Edoxaban)	Direct inhibitors of factor Xa (Apixaban, Rivaroxaban, Edoxaban) or thrombin (Dabigatran).	Dose depends on indication (e.g., apixaban 5 mg PO BID for VTE treatment).	Active bleeding, severe hepatic impairment, pregnancy, and mechanical heart valves.	May be used for post-discharge thromboprophylaxis in selected COVID-19 patients. Monitor renal and hepatic function.
Abatacept	Binds to the costimulatory molecules CD80 and CD86 on antigen-presenting cells (APC), thereby blocking interaction with CD28 on T cells.	0 mg/kg actual body Weight (up to 1,000 mg) administered as a single IV dose.	Check if you've previously been exposed to tuberculosis (TB). If the tests are positive, you may need to start a course of treatment for TB before beginning Abatacept.	No adjustment based on eGFR.
Fondaparinux	Anticoagulant, inhibitor of factor Xa and thrombin.	Dependent on the indication.	Creatinine clearance <30 mL/min, body Weight <50 kg, if used for VTE prophylaxis, active major bleeding, or bacterial endocarditis.	Regularly monitor signs and symptoms of neurologic impairment.
Sarilumab	Binds to membrane-bound and soluble IL-6 receptor forms, thus blocking the cis- and trans-inflammatory signaling cascades of IL-6.	Reconstitute sarilumab 400 mg in 100 cc 0.9% NaCl and administer as an IV infusion over 1 h.	Documented hypersensitivity to the drug or inactive ingredients.	Closely monitor for signs and symptoms of infection during treatment; if a severe infection develops, interrupt sarilumab therapy until the infection is controlled.
Recombinant tissue plasminogen activator (rt-PA)	Fibrinolytic agent, and fibrin-specific.	Dose dependent on the indication.	History of intracranial hemorrhage, intracranial neoplasm, or stroke within the last 3 months.	Regular monitoring of prothrombin time, aPTT, hemoglobin, and hematocrit.

aPTT, activated partial thromboplastin time; DIC, disseminated intravascular coagulation; eGFR, estimated glomerular filtration rate; IV, intravenous; PO, oral; RNA, ribonucleic acid; UFH, Unfractionated heparin.

#### 4.7.2 Management of venous thrombosis

##### 4.7.2.1 Deep vein thrombosis (DVT)

The management of deep vein thrombosis (DVT) in patients with COVID-19 remains complex due to the coexistence of a prothrombotic state and increased bleeding risk. According to the National Institute for Health and Care Excellence (NICE) guidelines, low-molecular-weight heparin (LMWH) is recommended for thromboprophylaxis and treatment in patients with a creatinine clearance above 30 mL/min. In contrast, a reduced LMWH dose or unfractionated heparin (UFH) 500 IU subcutaneously is advised in those with renal impairment (74). Recent clinical evidence suggests that intermediate or higher LMWH doses for VTE prophylaxis, indicating standard dose 40 mg every 24 h or increased dose >40 mg every 24 h, can be beneficial in preventing thrombotic events in severe COVID-19 cases, with a favorable balance between efficacy and bleeding risk when appropriately monitored (78–80).

As highlighted by Tudoran et al. (81), the coexistence of thrombosis and spontaneous hematomas during recovery illustrates the delicate balance between adequate anticoagulation and avoiding hemorrhage. Therefore, clinical decisions must weigh the bleeding risk, especially in elderly or comorbid patients, against the risk of thromboembolism by closely monitoring coagulation parameters, D-dimer levels, and platelet counts, which are crucial during treatment and recovery phases.

For this reason, patients with contraindications to anticoagulation, such as active bleeding or severe thrombocytopenia (platelet count <30,000/µL), mechanical thromboprophylaxis using graduated compression stockings or intermittent pneumatic compression is preferred. This personalized approach aims to reduce thrombotic risk while minimizing bleeding complications (79, 80).

For patients with confirmed or recurrent DVT, therapeutic anticoagulation with LMWH or UFH is recommended, followed by a switch to DOACs or vitamin K antagonists for at least three to six months, depending on clinical evolution and risk factors. The use of inferior vena cava (IVC) filters remains controversial and should be limited to patients with confirmed thromboembolism and absolute contraindications to anticoagulation, as their routine use has not demonstrated a mortality benefit (79, 80).

##### 4.7.2.2 Pulmonary embolism

Based on the American Society of Hematology guideline panel, prophylactic-intensity is preferred over therapeutic-intensity anticoagulation for patients with COVID-19-related critical illness without suspected or confirmed venous thromboembolism (VTE). In patients started on a therapeutic dose of heparin in a non-ICU setting and subsequently transferred to the ICU, a transition to a prophylactic heparin dose is suggested unless VTE is confirmed. Therefore, full therapeutic dose anticoagulation should be reserved for documented thromboembolic events, as its empirical use for thromboprophylaxis in critically ill patients may increase bleeding risk without improving survival (74, 82, 83).

The use of direct oral anticoagulants in severe COVID-19 has been supported by emerging evidence in severe COVID-19, particularly in stabilized patients transitioning from parenteral to oral therapy (15). In cases of persistent thromboembolic risk factors, such as IMPROVE scores of >4 or 2–3 times above D-dimer levels, normal levels, or without contraindications, should be treated with 10 mg once daily of Rivaroxaban (15).

The International Society on Thrombosis and Hemostasis recommends a prophylactic dose of unfractionated heparin (UFH) and low-molecular-weight heparin (LMWH) for non-critically ill hospitalized patients. Typically, 5,000 IU of UFH is given 2 or 3 times a day or 40 mg of LMWH once daily (84).

In critically ill patients requiring therapeutic anticoagulation, an initial bolus of unfractionated heparin (UFH) at 80 units/kg followed by a continuous infusion at 18 units/kg per hour is recommended, with the addition of tissue plasminogen activator (tPA) considered in cases of high-risk pulmonary embolism. Furthermore, patients in a non-ICU setting should be given Enoxaparin SC 1 mg per kg twice daily. If on direct oral anticoagulants, a switch to warfarin is recommended. Fondaparinux is the drug of choice for heparin-induced thrombocytopenia (15). For special populations, such as those with renal impairment, obesity (BMI >40 kg/m^2^), or pregnancy, UFH is preferred, as it does not require dose adjustment and has a safer profile in pregnancy (74).

**Figure 4:**
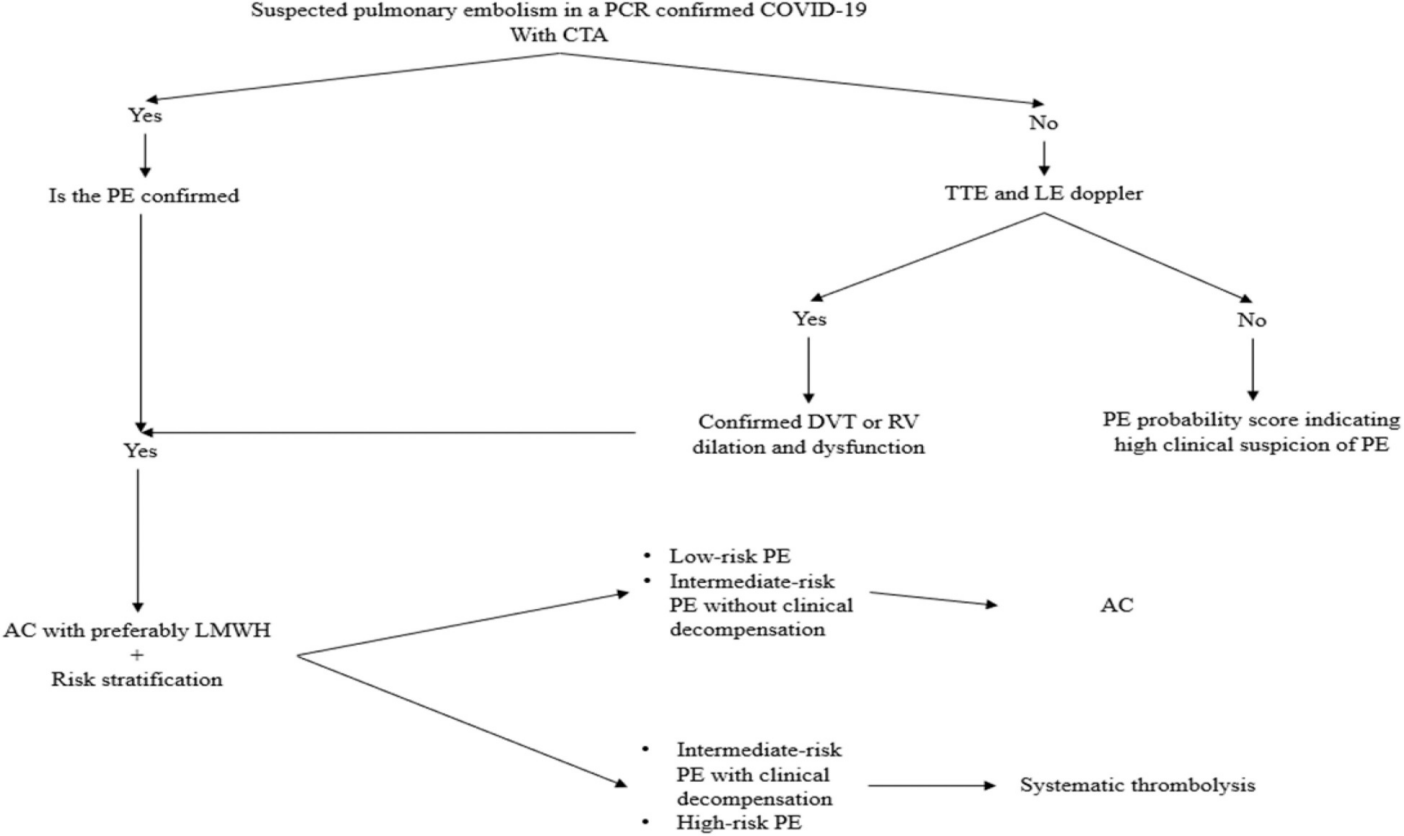
Diagnostic and management algorithm of pulmonary embolism during COVID-19. AC, anticoagulation; CTA, computed tomography angiography; DVT, deep vein thrombosis; LE, lower extremity; LMWH, low molecular weight heparin; PCR, polymerase chain reaction; PE, pulmonary embolism; RV, right ventricle; TTE, transthoracic echocardiogram.

Based on recommended practice, pharmacological and mechanical prevention methods should be used (70). Figure 4 illustrates the pulmonary embolism diagnosis and management algorithm during COVID-19.

This algorithm summarizes the evidence-based diagnostic and therapeutic pathway for managing pulmonary embolism (PE) in patients with confirmed COVID-19. It integrates recommendations from international societies, including the International Society on Thrombosis and Haemostasis (ISTH) (71) and the American Society of Hematology (ASH) (83).

#### 4.7.3 Management of arterial thrombosis

##### 4.7.3.1 Myocardial infarction

Myocardial infarction in patients with COVID-19 is increasingly recognized as a result of arterial thrombosis linked to inflammation, endothelial dysfunction, and coagulation abnormalities triggered by the infection. The management approach largely follows established acute coronary syndrome (ACS) protocols, yet requires certain modifications to address the unique thrombo-inflammatory environment of COVID-19 (85).

For patients presenting with ST-elevation myocardial infarction (STEMI), primary percutaneous coronary intervention (PCI) remains the treatment of choice whenever it can be performed promptly and safely. However, when timely transfer to a PCI facility is not feasible, fibrinolytic therapy remains a reasonable alternative, particularly for those at low procedural risk or in resource-limited centers. For non-ST-elevation myocardial infarction (NSTEMI), early invasive coronary angiography is typically reserved for unstable patients or those with a high Global Registry of Acute Coronary Events (GRACE) score >140. In contrast, stable patients may initially be managed conservatively until recovery from infection (73, 86–88).

Antithrombotic therapy forms the cornerstone of treatment and requires careful balancing between thrombotic and bleeding risks. Heparin (unfractionated or low-molecular-weight) and dual antiplatelet therapy (DAPT) are used according to current recommendations, with necessary dose adjustments in patients with renal impairment or concomitant anticoagulation. Given the endothelial injury and heightened platelet activation observed in COVID-19, maintaining optimal anticoagulant intensity is critical to prevent coronary re-thrombosis and microvascular obstruction (89, 90).

In severe cases complicated by cardiogenic shock or profound hypoxemia due to thrombotic microangiopathy, veno-arterial extracorporeal membrane oxygenation (VA-ECMO) may be required to support circulation and oxygenation. These patients pose particular management challenges, as inflammation and microthrombosis may limit reperfusion despite adequate intervention. Thus, a multidisciplinary approach involving cardiology, hematology, and critical care is often necessary to improve outcomes (91, 92).

In the context of COVID-19, myocardial infarction represents a complex interplay between inflammation, coagulation, and endothelial injury. Early recognition of thrombotic complications and tailored use of anticoagulant and antiplatelet therapy remain essential to reducing morbidity and mortality.

##### 4.7.3.2 Stroke

Ischemic stroke in patients with COVID-19 is increasingly recognized as a serious arterial thrombotic event linked to systemic inflammation, endothelial dysfunction, and hypoxia. A multicenter cohort study by Martí-Fàbregas et al. and another retrospective study reported that patients with COVID-19 experienced more severe strokes, with higher National Institutes of Health Stroke Scale (NIHSS) scores and worse functional outcomes compared with non-infected individuals (93, 94).

Intravenous thrombolytic therapy with tissue plasminogen activator (tPA) or Tenecteplase remains a practical option for eligible patients within the standard therapeutic window (≤4.5 h from symptom onset) and without contraindications. However, clinicians should carefully consider bleeding risk and potential hepatic dysfunction, which may alter drug clearance (95, 96).

For patients with large-vessel occlusion, mechanical thrombectomy remains the preferred treatment when feasible. Given the infection risk, inter-hospital transfers and procedural exposure should be minimized, and strict infection control precautions are recommended (97).

After reperfusion therapy, managing antithrombotic treatment is vital to prevent another ischemic event. Patients typically continue with antiplatelet drugs, or they may undergo dual antiplatelet therapy (DAPT) temporarily, depending on the stroke's cause and bleeding risk. If a cardioembolic source is found, anticoagulation is advised. During hospitalization, low-molecular-weight heparin (LMWH) is preferred because of its reliable pharmacokinetics, reduced monitoring needs, and lower likelihood of drug interactions compared to direct oral anticoagulants (93, 98).

#### 4.7.4 Drug–drug interactions in COVID-19–associated thrombosis management

In critically ill patients with COVID-19, managing anticoagulation remains a complex clinical decision due to the simultaneous presence of hypercoagulability and bleeding risk. Observational data from large tertiary centers have shown that thrombotic complications occur in about one-third of ICU patients. At the same time, major bleeding events affect about one-fifth, often associated with full-dose anticoagulation. These findings emphasize the importance of careful dose adjustment and individualized therapy, especially in patients with multiorgan dysfunction or elevated D-dimer levels (99, 100). A concise overview of these clinically relevant pharmacologic interactions is summarized in Table 2.

**Table 2:** Summary of key drug-drug interactions between COVID-19 and anti-thrombotic agents.

COVID-19 drug	Mechanism of interaction	Effect on anticoagulant/antiplatelet	Clinical manifestation
Ritonavir/Lopinavir	Potent CYP3A4 and P-gp inhibitors	↑ Plasma levels of DOACs (rivaroxaban, apixaban)	Higher bleeding risk; consider switching to LMWH or UFH during antiviral therapy
Dexamethasone	Moderate CYP3A4 inducer	↓ DOAC concentrations	Reduced anticoagulant efficacy, ↑ thrombotic risk; consider LMWH or dose monitoring
Remdesivir	Minimal CYP/P-gp effect	No major interaction reported	Continue anticoagulation as indicated
Tocilizumab	IL-6 inhibition restores CYP3A4 activity	May ↓ plasma concentration of drugs metabolized by CYP3A4	Clinically mild; monitor if combined with DOACs
Baricitinib	Immunomodulator (JAK inhibitor); possible additive bleeding risk	↑ Bleeding when combined with antiplatelets or heparins	Use with caution; monitor coagulation profile

In treating severe COVID-19, low-molecular-weight heparin (LMWH) and unfractionated heparin (UFH) are often preferred because they have predictable effects, can be easily reversed, and don't interact much with antiviral or immune-modulating drugs. While direct oral anticoagulants (DOACs) like Rivaroxaban and Apixaban are substrates of the cytochrome P450 3A4 (CYP3A4) system and P-glycoprotein (P-gp). It increases the risk of bleeding when used alongside antiviral medications such as Ritonavir or Lopinavir (101). Conversely, dexamethasone, a moderate CYP3A4 inducer, can lower DOAC levels, potentially reducing anticoagulant efficacy (13).

Remdesivir shows minimal pharmacokinetic interaction with anticoagulants because it is neither a significant substrate nor an inhibitor or inducer of CYP3A4 or P-gp (102). Nevertheless, clinicians should monitor hepatic enzymes, as elevated transaminases can occur with Remdesivir therapy, potentially complicating anticoagulant metabolism in patients with pre-existing liver dysfunction (103).

By contrast, tocilizumab, an IL-6 receptor blocker, can restore CYP3A4 activity suppressed by systemic inflammation, potentially increasing the metabolism of CYP3A4 substrates such as DOACs and thereby reducing their anticoagulant effect (104). In contrast, baricitinib may increase bleeding risk when combined with heparins or antiplatelet agents (105).

**Figure 5:**
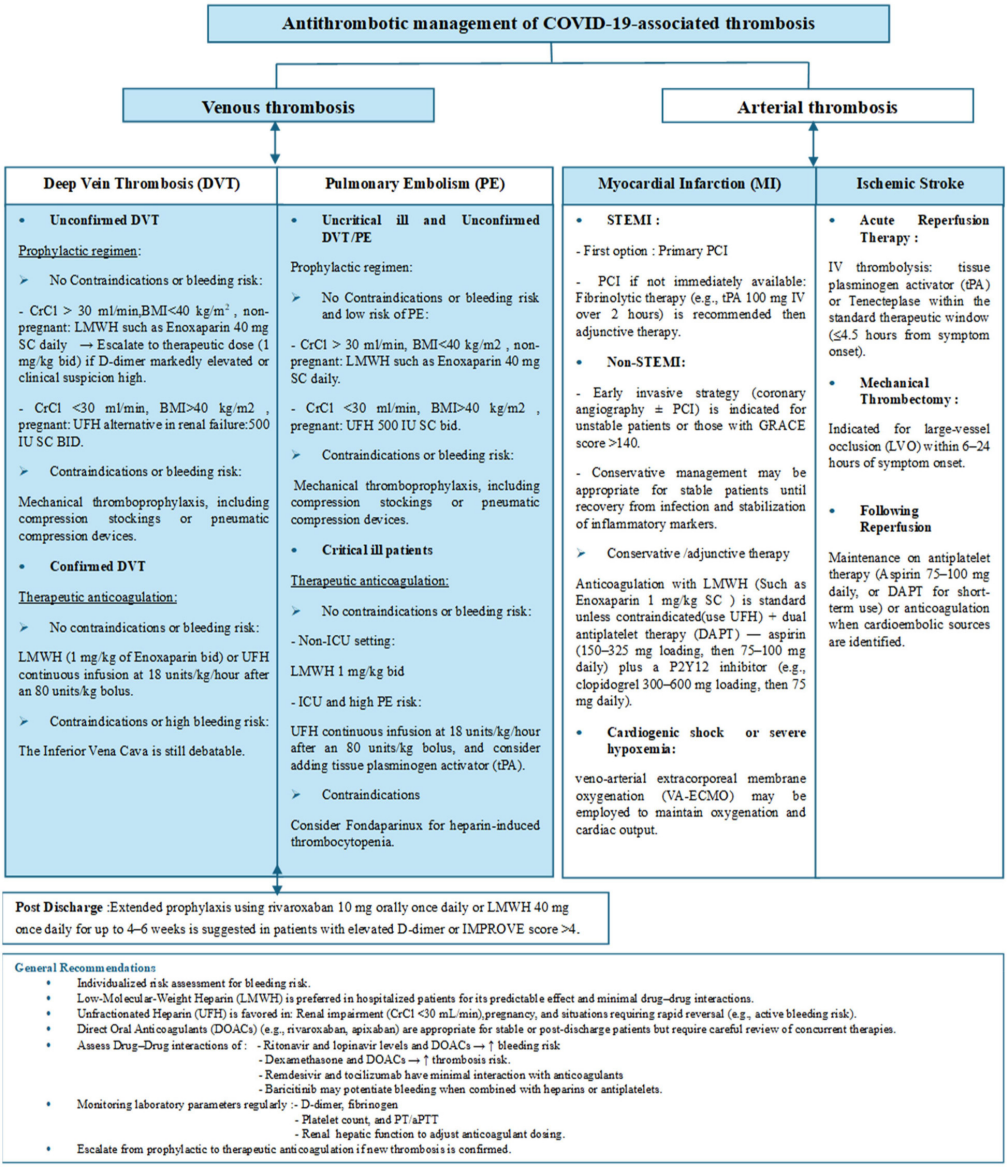
The major management protocols and pharmacologic recommendations for both venous and arterial thrombosis in COVID-19 patients. Crcl, creatinine clearance; BMI, body mass index; LMWH, low molecular weight heparin; UFH, unfractionated heparin; ICU, intensive care unit.

Due to these overlapping mechanisms, treatment choices should be guided by hepatic and renal function, concurrent medications, and current inflammatory status. Regular monitoring of coagulation parameters and periodic reassessment of therapy are essential to balance the risks of thrombosis, prevent adverse interactions, and improve safety. The choice of an anticoagulant should be based on the patient's clinical condition, liver and kidney function, and the specific antiviral or anti-inflammatory therapies being used. Figure 5 summarizes the major management protocols and recommendations for thrombosis in COVID-19.

## 5 Discussion

This scoping review synthesized evidence from 99 studies examining the pathogenesis, risk factors, and management of venous and arterial thrombosis in COVID-19. Studies show that when someone gets infected with SARS-CoV-2, their blood becomes more likely to form clots because of several factors, such as problems with the blood vessel lining, increased immune activity, and an imbalance in the clotting system. Thrombosis in veins and arteries shares similar pathways, most notably cytokine storm, the complement system activation, and injuries to blood vessel walls. Still, they also have key differences in how they form and affect the body. Clots in veins are mostly made up of fibrin and are linked to slow blood flow and widespread inflammation. In contrast, clots in arteries are mostly made up of platelets and are connected to inflammation of the blood vessel lining and overactive platelets (12, 31). These mechanistic distinctions reinforce the concept of immunothrombosis, linking immune dysregulation to vascular occlusion and multi-organ dysfunction in COVID-19 (9, 14).

Differences in study design, diagnostic criteria, and thromboprophylaxis protocols lead to significant variations in thrombotic complications among hospitalized COVID-19 patients. Venous thromboembolism (VTE) rates differ widely depending on the specific demographics of the hospitalized patient, with rates ranging from 3.9 to 14.7% in the general hospitalized population and reaching as high as 32.7% among intensive care patients, even with standard anticoagulation, as reported by Thomas and Scully et al. (2), Lillicrap (10), and Angélina et al. (17). Di Minno et al. (18) conducted a meta-analysis of 20 studies found that the pooled incidence of VTE was 31.3%, suggesting that conventional thromboprophylaxis was not effective. A similar pooled analysis by Liao et al. (56) and Roncon et al. (57) found that there is a high prevalence of pulmonary embolism (PE) among hospitalized patients, especially those with high D-dimer levels and severe inflammation. Despite being less common, arterial complications are equally severe. Myocardial injury has been reported in 25% of 50 hospitalized patients, and more than 50% of those with pre-existing cardiovascular conditions, as noted by Shi et al. (52), Martí-Fàbregas et al. (93), and Zhu et al. (106) have observed ischemic stroke in 1%–6%. These studies indicated how thromboinflammation contributes to the adverse outcomes in COVID-19.

The main reason for COVID-19-related thrombosis is endothelial dysfunction, as evidence indicates. SARS-CoV-2 infects endothelial cells via ACE2 receptors, leading to endothelialitis and the release of procoagulant factors (11, 31). The cytokine storm amplifies thrombin generation and platelet aggregation [Shu et al. (30); Levi et al. (32)]. Complement activation (33) and the formation of neutrophil extracellular traps further propagate vascular injury and coagulation (14, 34). Elevated fibrinogen, significantly increased D-dimer levels, and relatively normal platelet counts are hallmark features of COVID-19-associated coagulopathy, which differs from disseminated intravascular coagulation (10, 13, 107). These laboratory markers have become crucial for assessing risk and predicting outcomes in patients who are affected.

Anticoagulation continues to be the primary and essential approach in managing patients with COVID-19 in the treatment of thrombotic events, both in the veins and arteries. The use of low-molecular-weight heparin (LMWH) is preferred due to its predictable pharmacokinetics and anti-inflammatory properties, which have been shown to improve survival rates in severe diseases (70, 71). The HEP-COVID trial conducted by Spyropoulos et al. (71) demonstrated that therapeutic-dose heparin was superior to prophylactic dosing in decreasing thromboembolic events and death. However, bleeding risks remain non-negligible (38). Most hospitalized patients should receive anticoagulation at a prophylactic level, and the dose should be increased for those who are at a higher risk of developing blood clots, as recommended by the American Society of Hematology (ASH) and the International Society on Thrombosis and Haemostasis (ISTH) (82, 84). As per Dai et al. (77), prolonged post-discharge prophylaxis has been supported since it resulted in lower rates of thrombotic recurrence and mortality without any increase in bleeding.

Despite recent progress, significant gaps remain in understanding and managing COVID-19-related thrombosis. Most studies are retrospective and conducted at single centers, with inconsistent diagnostic criteria and anticoagulation protocols, making comparisons difficult (15, 17). Multicenter prospective trials are essential to define standard criteria for COVID-19-related coagulopathy, determine the most effective anticoagulant dose and duration, and evaluate long-term arterial outcomes (82, 84). Evidence of ongoing endothelial dysfunction and hypercoagulability after recovery raises concerns about chronic vascular complications and “long COVID” syndromes (6, 15). Furthermore, the impact of emerging SARS-CoV-2 variants, reinfections, and vaccine-induced immunity on thrombosis risk remains unclear (8, 37). Future research should integrate clinical, molecular, and imaging markers to assess individual risk better and improve prevention strategies for blood clots, supported by collaborative registries and adaptive clinical trials capable of adjusting to the evolving pandemic landscape.

This review has certain methodological limitations that come from its scoping approach. By focusing only on English-language publications, it may have missed out on regional data. Additionally, the various studies included made conducting a meta-analysis impossible. Since the COVID-19 literature is still growing and changing, some newer studies might not have been included. Despite these limitations, the review provides a broad look at the existing evidence and highlights important areas for future research.

## 6 Conclusion

The COVID-19 pandemic added some challenges in diagnosing and treating patients with thrombosis. Moderate to severe COVID-19 is associated with an inflammatory response that causes an acute-phase response and endothelial dysfunction, resulting in a hypercoagulable state known as COVID-19-associated coagulopathy. Guidelines were modified to provide patients with the best possible medical care in a controlled environment that reduces the spread of COVID-19 infection. Anticoagulants should be prescribed to hospitalized COVID-19 patients to prevent thrombosis, including antithrombotic prophylaxis that should be applied to all patients, in contrast to therapeutic anticoagulation given only to critically ill patients with confirmed venous thromboembolism. In some instances, tPA protocol is also required. COVID-19 patients are treated per protocol; however, thoughtful assessment of risks and benefits is done regarding drug-drug interaction, transfer to another hospital, and post-discharge therapy. Selection of the appropriate medication depends on the disease, medical tests, and co-existing diseases.
