# Supplementary material for: Scoping review about pathogenesis, risk factors, and treatment of venous and arterial thrombosis in coronavirus infection
Source: Front Cardiovasc Med. 2025 Dec 12;12:1688115. doi: 10.3389/fcvm.2025.1688115 (PMC12741095; doi:10.3389/fcvm.2025.1688115)
Supplement: Supplementary file 1 [file Table1.docx]

**Supplementary files**

| Author (Year) | Title | Country / Setting | Study Design | Aim |
| --- | --- | --- | --- | --- |
| Tian D et al. (2021)(8) | The Global Epidemic of the SARS-CoV-2 Delta Variant, Key Spike Mutations and Immune Escape | Global | Narrative review | Summarize Delta spike mutations, spread, and immune escape. |
| Sutanto H, Soegiarto G (2023)(22) | Risk of Thrombosis during and after a SARS-CoV-2 Infection: Pathogenesis, Diagnostic Approach, and Management | Global | Narrative review | Review thrombosis risk during/after infection and clinical management. |
| Ackermann M et al. (2020)(9) | Pulmonary Vascular Endothelialitis, Thrombosis, and Angiogenesis in Covid-19 | Germany/USA (labs) | Pathology (autopsy + comparison) | Describe Endothelialitis, thrombosis, and angiogenesis in fatal COVID-19 lungs. |
| Nalbandian A et al. (2021)(6) | Post-acute COVID-19 syndrome | Global | Narrative review | Synthesize evidence on long COVID manifestations and mechanisms. |
| Wiersinga WJ et al. (2020)(4) | Pathophysiology, Transmission, Diagnosis, and Treatment of COVID-19: A Review | Global | Narrative review | Comprehensive review of biology, diagnosis, and treatment. |
| Middleton EA et al. (2020)(110) | Neutrophil extracellular traps contribute to immunothrombosis in COVID-19 ARDS | USA | Translational/mechanistic study | Demonstrate NETs’ role in immunothrombosis in COVID-19 ARDS. |
| Portier I, Campbell RA, Denorme F (2021)(14) | Mechanisms of immunothrombosis in COVID-19 | Global | Narrative review | Summarize immune–coagulation mechanisms driving thrombosis. |
| Tudoran C et al. (2021)(7) | Factors Influencing the Evolution of Pulmonary Hypertension in Previously Healthy Subjects Recovering from SARS-CoV-2 | Romania | Cohort study | Identify determinants of post-COVID pulmonary hypertension. |
| Gupta A et al. (2020)(37) | Extrapulmonary manifestations of COVID-19 | Global | Narrative review | Summarize systemic manifestations beyond the lungs. |
| Bonaventura A et al. (2021)(12) | Endothelial dysfunction and immunothrombosis as key pathogenic mechanisms in COVID-19 | Global | Narrative review | Frame endothelial dysfunction and immunothrombosis as central. |
| Varga Z et al. (2020)(11) | Endothelial cell infection and endotheliitis in COVID-19 | Switzerland | Pathology/case series | Report endothelial infection/endotheliitis with histology/EM. |
| Lillicrap D (2020)(10) | Disseminated intravascular coagulation in patients with 2019‑nCoV pneumonia | Global | Narrative review/commentary | Discuss DIC in the COVID-19 context. |
| Angelini DE et al. (2022)(17) | COVID-19 and venous thromboembolism: A narrative review | Global | Narrative review | Summarize VTE epidemiology and management in COVID-19. |
| Di Minno A et al. (2020)(18) | COVID-19 and Venous Thromboembolism: A Meta-analysis of Literature Studies | Global | Systematic review & meta-analysis | Quantify VTE rates in COVID-19. |
| Connors JM, Levy JH (2020)(13) | COVID-19 and its implications for thrombosis and anticoagulation | Global | Narrative review/expert perspective | Summarize thrombotic risk and anticoagulation strategies. |
| World Health Organization (2024)(1) | Coronavirus Dashboard | Global | Surveillance dashboard | Provide global situation metrics for COVID-19. |
| Levi M et al. (2020)(33) | Coagulation abnormalities and thrombosis in patients with COVID-19 | Global | Narrative review | Review coagulopathy and thrombosis pathogenesis/management. |
| Thomas, MR, Scully, M (2022)(2) | Clinical features of thrombosis and bleeding in COVID-19 | Global | Narrative review | Summarize thrombotic and bleeding presentations and risks. |
| Arentz M et al. (2020)(16) | Characteristics and Outcomes of 21 Critically Ill Patients With COVID-19 in Washington State | USA | Case series | Describe features/outcomes of the early critically ill cohort. |
| Zhu N et al. (2020)(3) | A Novel Coronavirus from Patients with Pneumonia in China, 2019 | China | Virology/identification study | Identify and characterize SARS‑CoV‑2 from patient samples. |
| Rosovsky RP et al. (2020)(111) | Diagnosis and treatment of pulmonary embolism during the COVID-19 pandemic: PERT position paper | USA (guidance) | Position paper/consensus | Provide PE diagnosis/treatment guidance during the pandemic. |
| Cheng C et al. (2021)(20) | The incubation period of COVID-19: a global meta-analysis and Chinese observation | Global + China | Systematic review and meta-analysis (SR/MA) with original cohort data | Estimate the incubation period from the literature and 11,545 Chinese patients. |
| Pan X et al. (2020)(21) | Asymptomatic cases in a family cluster with SARS-CoV-2 infection | China | Case series | Describe asymptomatic transmission in a family cluster. |
| Danzi GB et al. (2020)(24) | Acute pulmonary embolism and COVID‑19 pneumonia: a random association? | Italy | Case report/short series | Report on PE occurrence in COVID-19 pneumonia. |
| Linton NM et al. (2020)(25) | Incubation Period and Other Epidemiological Characteristics... with Right Truncation | Global | Statistical/methods analysis | Estimate incubation/epidemiologic parameters using public data. |
| Zhou F et al. (2020)(23) | Clinical course and risk factors for mortality of adult inpatients with COVID-19 in Wuhan | China (Wuhan) | Retrospective cohort | Identify mortality risk factors and clinical course. |
| Lippi G et al. (2020)(27) | Clinical and demographic characteristics of patients dying from COVID‑19 in Italy vs China | Italy & China | Comparative epidemiology | Compare decedent characteristics between countries. |
| AlAmri AS et al. (2025)(28) | Arteriovenous Thrombosis among SARS‑CoV‑2 Infected Patients: Observational Study | Saudi Arabia | Observational cohort | Assess the incidence of arterial/venous thrombosis in hospitalized patients. |
| Velissaris D et al. (2023)(29) | Anticoagulation in COVID‑19: pathophysiology, clinical use, and dosing | Global | Narrative review | Review pathophysiology and optimal anticoagulation dosing in/outpatient. |
| Ortega-Paz L et al. (2023)(30) | COVID‑19-Associated Pulmonary Embolism: Review | Global | Narrative review | Summarize PE pathophysiology, epidemiology, prevention, diagnosis, and treatment. |
| Shu H et al. (2023)(31) | Understanding COVID‑19-related myocarditis | Global | Narrative review | Summarize myocarditis mechanisms, diagnosis, and treatment in COVID-19. |
| Conway EM et al. (2022)(32) | Understanding COVID‑19-associated coagulopathy | Global | Narrative review | Integrate current understanding of COVID‑19 coagulopathy. |
| Levi M et al. (2020)(33) | Coagulation abnormalities and thrombosis in patients with COVID‑19 | Global | Narrative review | Duplicate of #18. |
| Lim EHT et al. (2023)(35) | Complement activation in COVID‑19 and targeted therapeutic options: A scoping review. | Global | Scoping review | Map complement activation evidence and therapies. |
| Klok FA et al. (2020)(38) | Incidence of thrombotic complications in critically ill ICU patients with COVID‑19 | Netherlands | Retrospective/prospective cohort | Estimate thrombotic complication incidence in ICU patients. |
| Bekal S et al. (2023)(39) | Thrombosis Development After mRNA COVID‑19 Vaccine Administration: A Case Series | USA | Case series | Describe thrombosis events following mRNA vaccination. |
| Lee H‑J et al. (2023)(40) | Therapeutic or intermediate vs prophylactic anticoagulation in COVID‑19: SR/MA | Global | Systematic review & meta-analysis | Compare efficacy/safety across anticoagulation intensities. |
| El‑Hady HA et al. (2023)(41) | Portal vein thrombosis in patients with COVID‑19: A systematic review | Global | Systematic review | Summarize PVT cases and outcomes in COVID-19. |
| Bikdeli B et al. (2020)(42) | COVID‑19 and Thrombotic/Thromboembolic Disease: JACC State‑of‑the‑Art Review | Global | Narrative review | Guidance for prevention, therapy, and follow‑up of thrombotic disease. |
| Sethi SM et al. (2022)(43) | Association of thrombosis and mortality in patients with COVID‑19 infections | Egypt | Observational hospital cohort | Examine the link between thrombosis and mortality. |
| Biswas S et al. (2021)(44) | Blood clots in COVID‑19 patients: Simplifying the curious mystery | Global | Hypothesis/commentary | Propose mechanisms and conceptual framing for clotting. |
| Atri D et al. (2020)(45) | COVID‑19 for the Cardiologist: Basic Virology, Epidemiology, Cardiac Manifestations, and Strategies | Global | Narrative review | Summarize cardiac aspects and potential therapies. |
| Del Prete A et al. (2022)(46) | COVID‑19, Acute Myocardial Injury, and Infarction | Global | Narrative review | Review myocardial injury/infarction related to COVID‑19. |
| Zhu Y et al. (2022)(47) | Consequences of COVID‑19 on the cardiovascular and renal systems | Global | Narrative review | Summarize CV and renal consequences of COVID‑19. |
| Pirzada A et al. (2020)(48) | COVID‑19 and Myocarditis: What Do We Know So Far? | Global | Narrative review | Summarize evidence on myocarditis in COVID‑19. |
| Guagliumi G et al. (2020)(50) | Microthrombi and ST‑Elevation MI in COVID‑19 | Italy | Case series/pathology correlation | Describe coronary microthrombi associated with STEMI in COVID‑19. |
| Ortega‑Paz L et al. (2020)(51) | Coronavirus Disease 2019‑Associated Thrombosis and Coagulopathy | Global | Narrative review | Review pathophysiology & antithrombotic management. |
| Corrales‑Medina VF et al. (2015)(52) | Pneumonia hospitalization and subsequent cardiovascular risk | USA | Retrospective cohort | Assess long‑term CVD risk after pneumonia hospitalization. |
| Chapman AR et al. (2018)(55) | Long‑Term Outcomes in Type 2 MI and Myocardial Injury | UK | Observational cohort | Describe outcomes for type 2 MI/myocardial injury. |
| Shi S et al. (2020)(54) | Association of Cardiac Injury With Mortality in Hospitalized COVID‑19 | China (Wuhan) | Retrospective cohort | Evaluate cardiac injury in relation to mortality. |
| Lakkireddy DR et al. (2020)(56) | Guidance for cardiac electrophysiology during the COVID‑19 pandemic | USA (HRS/ACC/AHA) | Guideline/consensus | Operational guidance for EP care. |
| Wang Q et al. (2024)(57) | Impact of COVID‑19 on prognosis of DVT after anticoagulation: two‑year cohort | China | Retrospective cohort (single‑center) | Assess outcomes of DVT patients with/without COVID‑19. |
| Liao S‑C et al. (2020)(58) | Incidence and mortality of pulmonary embolism in COVID‑19: SR/MA | Global | Systematic review & meta-analysis | Pool PE incidence and mortality estimates. |
| Roncon L et al. (2020)(59) | Incidence of acute pulmonary embolism in COVID‑19 patients: SR/MA | Global | Systematic review & meta-analysis | Estimate acute PE incidence in COVID‑19. |
| Riyahi S et al. (2021)(60) | Pulmonary Embolism in Hospitalized Patients with COVID‑19: A Multicenter Study | USA | Multicenter observational | Determine pulmonary embolism prevalence and predictors in hospitalized patients. |
| Scudiero F et al. (2021)(61) | Pulmonary embolism in COVID‑19 patients: prevalence, predictors, and outcome | Italy | Observational cohort | Assess pulmonary embolism prevalence, predictors, and outcomes. |
| Engelen MM et al. (2021)(62) | Venous Thromboembolism in Patients Discharged after COVID‑19 Hospitalization | Global | Narrative review | Summarize post‑discharge Venous thromboembolism risk and evidence. |
| Barda N et al. (2021)(63) | Safety of the BNT162b2 mRNA COVID-19 Vaccine in a Nationwide Setting | Israel | Nationwide matched cohort | Assess the safety signals of the BNT162b2 vaccine. |
| Wada N et al. (2023)(64) | Incidence and severity of pulmonary embolism in COVID‑19 infection: Ancestral, Alpha, Delta, Omicron | Japan | Retrospective cohort | Compare pulmonary embolism across variant waves. |
| Agudo M et al. (2023)(65) | Pulmonary Embolism of COVID‑19: A Year of Reflection | Portugal | Narrative review | Reflect on pulmonary embolism management and cases over a year. |
| Mouzarou A et al. (2022)(14) | Pulmonary Embolism in Post‑COVID‑19 Patients: A Literature Review | Global | Narrative review | Summarize pulmonary embolism after recovery from COVID‑19. |
| Demelo‑Rodríguez P et al. (2020)(67) | Incidence of asymptomatic DVT in COVID‑19 pneumonia with elevated D‑dimer | Spain | Prospective screening cohort | Screen for asymptomatic DVT in hospitalized patients. |
| Zhang et al. (2020)(68) | Correction to: Deep Vein Thrombosis in Hospitalized Patients With COVID‑19 in Wuhan, China | China (orig.) | Correction notice | Correct the earlier prevalence report. |
| Cai C et al. (2020)(69) | Deep Venous Thrombosis in COVID‑19 Patients: A Cohort Analysis | China | Cohort study | Assess deep vein thrombosis prevalence, risk factors, and outcomes. |
| Suthar AB et al. (2022)(70) | Coronavirus Disease Case Definitions, Diagnostic Testing Criteria, and Surveillance in 25 Countries with the Highest Reported Case Count | Multi‑country | Comparative policy analysis | Compare surveillance definitions/testing across countries. |
| Yaghi et al. (2020)(100) | SARS-CoV-2 and Stroke in a New York Healthcare System | USA (New York, multicenter health system) | Observational cohort study | To investigate the incidence, clinical characteristics, and outcomes of acute ischemic stroke among patients with COVID-19 within an extensive New York healthcare network. |
| Furnica C et al. (2022)(75) | Impact of Early Pandemic on STEMI Presentation and Outcomes—SR/MA | Global | Systematic review & meta-analysis | Assess changes in STEMI care/outcomes during the early pandemic. |
| NIH (2025)(76) | COVID‑19 Treatment Guidelines (Accessed) | USA (guideline site) | Guideline resource | Provide current treatment recommendations. |
| Vincent J‑L, Levi M, Hunt BJ (2022)(72) | Prevention and management of thrombosis in hospitalized COVID‑19 pneumonia | Global | Review/guidance | Summarize prevention/management recommendations. |
| Xie Y et al. (2023)(77) | Nirmatrelvir and the Risk of Post‑COVID‑19 Condition | USA | Observational cohort ( | Evaluate the association of nirmatrelvir with long‑COVID risk. |
| Szilveszter M et al. (2023)(74) | Management of COVID‑19‑Related Coagulopathy: Metabolic & Vascular Disease Challenges | Global | Narrative review | Discuss coagulopathy management with comorbidities. |
| RECOVERY Collaborative Group (2021)(78) | Dexamethasone in Hospitalized Patients with Covid‑19 | UK | Randomized controlled trial | Test the effect of dexamethasone on mortality. |
| Dai M‑F et al. (2023)(79) | Extended thromboprophylaxis post‑discharge in COVID‑19: SR/MA | Global | Systematic review & meta-analysis | Evaluate efficacy/safety of extended thromboprophylaxis. |
| Szerlip M et al. (2020)(89) | Cath lab procedures during COVID‑19—SCAI ELM perspectives | USA | Consensus/position paper | Operational considerations for the cath lab during the pandemic. |
| Zeng J et al. (2020)(91) | Balancing acute myocardial infarction and COVID‑19: protocols from Sichuan Provincial People’s Hospital | China | Protocol/experience report | Describe acute myocardial infarction care protocols during the COVID‑19 surge. |
| Guddeti RR et al. (2023)(87) | Impact of COVID‑19 on Acute Myocardial Infarction Care | Global | Narrative review | Summarize system‑level and clinical effects on AMI care. |
| Lemkes JS et al. (2019)(112) | Coronary Angiography after Cardiac Arrest without ST‑Elevation | Netherlands | Randomized controlled trial | Test routine angiography vs delayed/selective approach. |
| Rab T et al. (2015)(93) | Cardiac arrest: Algorithm for emergent invasive procedures in a resuscitated comatose patient | USA | Expert consensus/algorithm | Propose an invasive management algorithm post–cardiac arrest. |
| Wang Y et al. (2022)(90) | STEMI with vs without COVID‑19: Systematic Review and Meta‑Analysis | Global | Systematic review & meta-analysis | Compare characteristics, management, and outcomes. |
| Cuker A et al. (2021)(84) | ASH 2021 guidelines on thromboprophylaxis in patients with COVID‑19 | Global (ASH/USA) | Guideline | Recommend anticoagulation strategies for different severities. |
| Cuker A et al. (2022)(85) | ASH living guidelines: Jan 2022 update on therapeutic‑intensity anticoagulation | Global (ASH/USA) | Guideline update | Update on use of therapeutic‑intensity anticoagulation. |
| Schulman S et al. (2022)(86) | ISTH guidelines for antithrombotic treatment in COVID‑19 | Global | Guideline | Provide ISTH recommendations for antithrombotic therapy. |
| Günertem E (2020)(81) | Treatment and prophylaxis strategies for DVT during the COVID‑19 outbreak | Turkey/Global | Narrative review | Outline DVT strategies tailored to the pandemic context. |
| French J, Greenwood K (2023)(82) | LMWH doses for VTE prophylaxis in COVID‑19: evidence review | UK/Global | Evidence review | Compare prophylactic dosing regimens and outcomes. |
| Martí‑Fàbregas J et al. (2021)(95) | Impact of COVID‑19 Infection on the Outcome of Patients With Ischemic Stroke | Spain | Observational registry/cohort | Assess outcomes of ischemic stroke patients with COVID‑19. |
| Amiri HA et al. (2022)(96) | Effects of COVID‑19 on Patients with Acute Ischemic and Hemorrhagic Stroke | Iran | Observational cohort | Compare characteristics/outcomes in stroke patients by COVID status. |
| Khimani F et al. (2023)(97) | Therapeutic considerations for prevention and treatment of thrombotic events in COVID‑19 | Global | Narrative review | Summarize prevention and treatment strategies for thrombosis. |

Table S1. Included Study characteristics.
